# Supplementary material for: Turn-on chemiluminescence probes and dual-amplification of signal for detection of amyloid beta species in vivo
Source: Nat Commun. 2020 Aug 13;11:4052. doi: 10.1038/s41467-020-17783-4 (PMC7426431; doi:10.1038/s41467-020-17783-4)
Supplement: Supplementary file 1 — Supplementary Information [file 41467_2020_17783_MOESM1_ESM.pdf]

Supplementary Information

Turn-on chemiluminescence probes and dual-amplification of signal for detection of amyloid beta species in vivo

Yang et al.

Supplementary Methods.

Reagents used for the synthesis were purchased from Sigma-Aldrich and used without further purification. Column chromatography was performed on a glass column slurry-packed with silica gel (60 Å, 40–63 mm; SiliCycle Inc.). Recombinant Aβ peptide (1-40/42) were purchased from rPeptide (A-1163-1). Aβ aggregates for in vitro studies were generated by slow stirring of Aβ40 in PBS buffer for 3 days at room temperature. ^{1}H and^{13}C NMR spectra were recorded at 500 MHz and 125 MHz on Bruker spectrometers in $CDCl_3$, CD_3OD or DMSO-d_6 solutions at room temperature with tetramethylsilane (TMS, $\delta = 0$) as an internal standard. Liquid chromatography-mass spectrometry (LC-MS) was performed using an Agilent 1200 Series apparatus with an LC/MSD trap and Daly conversion dynode detector with UV detection at 254 nm. Fluorescence measurements were carried out using an F-7100 fluorescence spectrophotometer (Hitachi). Transgenic female 5xFAD mice and age-matched wild-type female mice were purchased from Jackson Laboratory. All animal experiments were approved by the Institutional Animal Use and Care Committee at Massachusetts General Hospital. Animals were housed in group in standardized cages with a 12/12 h light/dark cycle with unrestricted access to food and water at room temperature (ca. 25°C) with ca. 55% humidity. The IVIS Spectrum animal imaging system (PerkinElmer) was used for in vitro and vivo imaging.

Typical procedure for preparation of 1,2-disubtituted vinyl boronates. To a solution of TMP (2,2,6,6-Tetramethylpiperidine, 0.25 mmol, 1.0 equiv) in anhydrous THF (1.0 mL), *n*-BuLi (0.25 mmol, 1.0 equiv) was added at 0 °C. The resulting mixture was stirred for 5 minutes at 0 °C, followed by an addition of a solution of bis-(4,4,5,5-tetramethyl-1,3,2-dioxaborolan-2-yl)methane (0.30 mmol, 1.2 equiv) in THF (0.60 mL). The resulting solution was stirred at 0 °C for 15 minutes. Then the reaction vial was cooled to -78 °C, and a solution of aldehyde (0.25 mmol, 1.0 equiv) in THF (0.30 mL) was added. The reaction vial was stirred at -78°C for additional 4 hours. Upon completion, the reaction mixture was concentrated under reduced pressure and the 1,2-disubtituted-vinyl boronate products were purified by flash silica chromatography. *Compound **2a*** was obtained as a yellow solid, 72% yield. ^{1}H NMR (500 MHz, $CDCl_3$): δ 7.32 (d, J = 8.8 Hz, 2H), 7.20 – 7.14 (m, 1H), 6.70 – 6.59 (m, 4H), 5.55 (d, J = 17.5 Hz, 1H), 2.97 (s, 6H), 1.29 (s, 12H). ^{13}C NMR (125 MHz, $CDCl_3$): δ 150.93, 150.53, 136.86, 128.20, 126.51, 125.09, 112.28, 83.12, 40.43, 24.89. ESI-MS (m/z): 300.1 $[M+H]^+$.

*N,N-dimethyl-4-((1E,3E,5E)-6-(4,4,5,5-tetramethyl-1,3,2-dioxaborolan-2-yl)hexa-1,3,5-trien-1-yl)aniline (**2b**)*. Compound ***2b*** was prepared by the same method as **2a**. Yellow solid, 65% yield. ^{1}H NMR (500 MHz, $CDCl_3$): δ 7.29 (d, *J* = 8.7 Hz, 2H), 7.07 (dd, *J* = 17.5, 10.7 Hz, 1H), 6.68 – 6.60 (m, 3H), 6.57 – 6.46 (m, 2H), 6.32 (dd, *J* = 14.6, 10.8 Hz, 1H), 5.51 (d, *J* = 17.5 Hz, 1H), 2.96 (s, 6H), 1.26 (s, 12H). ^{13}C NMR (125 MHz, $CDCl_3$): δ 150.28, 137.83, 135.32, 132.54, 127.91, 125.58, 124.63, 112.43, 83.24, 40.51, 24.93. ESI-MS (m/z): 326.3 $[M+H]^+$.

*Synthesis of 5-((1E,3E)-4-(4-(dimethylamino)phenyl)buta-1,3-dien-1-yl)pyrazin-2-amine (**3a**):* Under an argon atmosphere, compound **2a** (106.0 mg, 0.39 mmol) and 5-bromo-2-aminopyrazine (57.0 mg, 0.33 mmol) were dissolved in a mixture of 1,4-dioxane (6.0 mL), and 1.0 M Na_2CO_3 aqueous solution (400.0 μL), and the resulting mixture was degassed in vacuo. A catalytic amount of tetrakis(triphenylphosphine)palladium(0) (0.0033 mmol, 3.8 mg) was added into the mixture and the mixture was heated at 75 °C for 6 h. After cooling, the mixture was diluted with EtOAc (20 mL) and washed with water and brine, dried over Na_2SO_4 and evaporated. The resulting residue was purified by flash silica chromatography (silica gel, eluent: chloroform /methanol = 50/1) to obtain **3a** as a yellow solid (56.0 mg, 64.0 %).^{1}H NMR (500 MHz, DMSO-d_6): δ 7.93 (s, 1H), 7.87 (s, 1H), 7.32 (d, *J* = 8.7 Hz, 2H), 7.10 (dd, *J* = 15.2, 10.9 Hz, 1H), 6.81 (dd, *J* = 15.4, 11.0 Hz, 1H), 6.69 (d, *J* = 8.8 Hz, 2H), 6.59 (d, *J* = 15.5 Hz, 1H), 6.53 – 6.50 (m, 3H), 2.92 (s, 6H). ^{13}C NMR (125 MHz, DMSO-d_6): δ 154.43, 149.92, 140.65, 139.02, 132.97, 132.18, 128.81, 127.42, 126.80, 125.21, 124.78, 112.32, 40.03. ESI-MS (m/z): 267.1 $[M+H]^+$.

*Synthesis of 5-((1E,3E,5E)-6-(4-(dimethylamino)phenyl)hexa-1,3,5-trien-1-yl)pyrazin-2-amine (**3b**)*. Compound ***3b*** was prepared by the same method as **3a**. ^{1}H NMR (500 MHz, DMSO-d_6): δ 7.92 (s, 1H), 7.87 (s, 1H), 7.31 (d, *J* = 8.7 Hz, 2H), 7.05 (dd, *J* = 15.2, 10.6 Hz, 1H), 6.75 (dd, *J* = 15.2, 10.6 Hz, 1H), 6.68 (d, *J* = 8.8 Hz, 2H), 6.58 – 6.40 (m, 6H), 2.92 (s, 6H). ^{13}C NMR (125 MHz, DMSO-d_6): δ 154.43, 149.87, 140.94, 138.72, 134.29, 132.83, 132.24, 131.05, 128.19, 127.66, 127.41, 125.10, 124.81, 112.25, 39.94. ESI-MS (m/z): 293.1 $[M+H]^+$.

*Synthesis of 6-((1E,3E)-4-(4-(dimethylamino)phenyl)buta-1,3-dien-1-yl)-2-methylimidazo[1,2-a]pyrazin-3(7H)-one (**ADLumin-1**)*. Under an argon atmosphere, compound **3a** (50.0 mg, 0.18

mmol) and methylglyoxal-1,1-dimethyl acetal (30.0 mg, 0.25 mmol) were dissolved in a mixture of 20% HCl aq (200.0 µL), water (200.0 µL), and EtOH (3.0 mL), and the resulting mixture was stirred and heated at 70 °C for 12 h. After cooling, the reaction mixture was evaporated. The resulting residue was purified by flash silica chromatography (eluent: Dichloromethane /methanol = 10/1) to obtain **ADLumin-1** as a yellow solid (10.0 mg, 17 %).^{1}H NMR (500 MHz, CD_3OD): δ 9.03 (s, 1H), 8.39 (s, 1H), 7.76 – 7.64 (m, 4H), 7.55 (t, *J* = 15 Hz, 1H), 7.23 (t, *J* = 15 Hz, 1H), 6.95 – 6.87 (m, 2H), 3.29 (s, 6H), 2.52 (s, 3H). ^{13}C NMR (125 MHz, CD_3OD): δ 143.14, 140.61, 139.91, 139.03, 135.88, 135.47, 135.14, 131.73, 129.69, 128.56, 127.26, 124.46, 122.22, 113.97, 47.2, 10.17. ESI-MS (m/z): 321.1 $[M+H]^+$. HR-MS (m/z): 321.1710 $[M+H]^+$.

*Synthesis of 6-((1E,3E,5E)-6-(4-(dimethylamino)phenyl)hexa-1,3,5-trien-1-yl)-2-methylimidazo [1,2-a]pyrazin-3(7H)-one (**ADLumin-2**).* **ADLumin-2** was prepared by the same method as **ADLumin-1**. Red solid, 12% yield. ^{1}H NMR (500 MHz, CD_3OD): δ 8.83 (s, 1H), 8.18 (s, 1H), 7.61 (s, 4H), 7.29 (dd, *J* = 14.8, 8.9 Hz, 1H), 6.99 (dd, *J* = 14.8, 8.6 Hz), 6.71 – 6.55 (m, 4H), 3.23 (s, 6H), 2.40 (s, 3H). ^{13}C NMR (125 MHz, CD_3OD): δ 142.87, 140.75, 137.76, 135.39, 134.79, 134.59, 133.27, 132.46, 129.73, 129.40, 128.57, 125.65, 122.10, 113.43, 47.15, 10.66. ESI-MS (m/z): 347.3 $[M+H]^+$. HR-MS (m/z): 347.1870 $[M+H]^+$.

*Synthesis of N-(5-((1E,3E)-4-(4-(dimethylamino)phenyl)buta-1,3-dien-1-yl)pyrazin-2-yl)acetamide (**ADLumin-3**).* To a solution of **ADLumin-1** (5 mg, 0.015 mmol) in DMSO (4 mL) was bubbled oxygen for 12 h. The mixture was diluted with EtOAc (15 mL) and washed with water and brine, dried over Na_2SO_4 and evaporated. The resulting residue was purified by flash silica chromatography (silica gel, eluent: Hexane/EtOAc = 2/1) to obtain **ADLumin-3** as a yellow solid (8 mg, 85%). ^{1}H NMR (500 MHz, DMSO-d_6): δ 10.75 (s, 1H), 9.23 (s, 1H), 8.42 (s, 1H), 7.42 – 7.36 (m, 3H), 6.90 (dd, *J* = 15.4, 10.9 Hz, 1H), 6.77 – 6.64 (m, 4H), 2.94 (s, 6H), 2.12 (s, 3H).^{13}C NMR (125 MHz, DMSO-d_6): δ 169.22, 150.23, 146.53, 146.31, 140.55, 135.95, 135.47, 133.85, 127.85, 125.27, 124.57, 123.92, 112.15, 39.85, 23.64. ESI-MS (m/z): 309.1 $[M+H]^+$.

Supplementary Figures

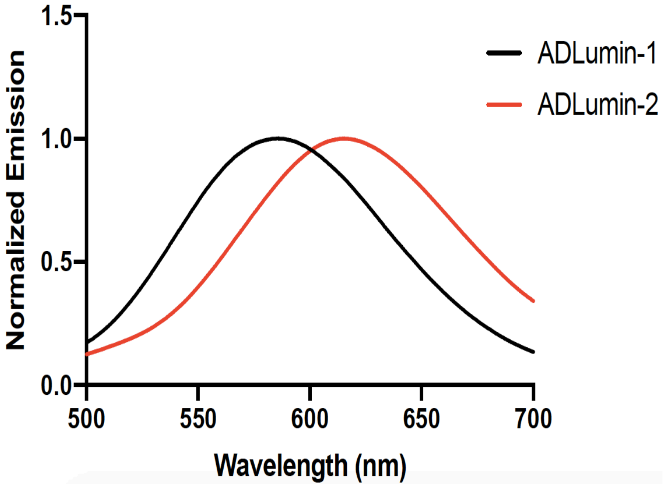

Supplementary Figure 1 Fluorescence emission spectra of ADLumin-1 and ADLumin-2 in DMSO.

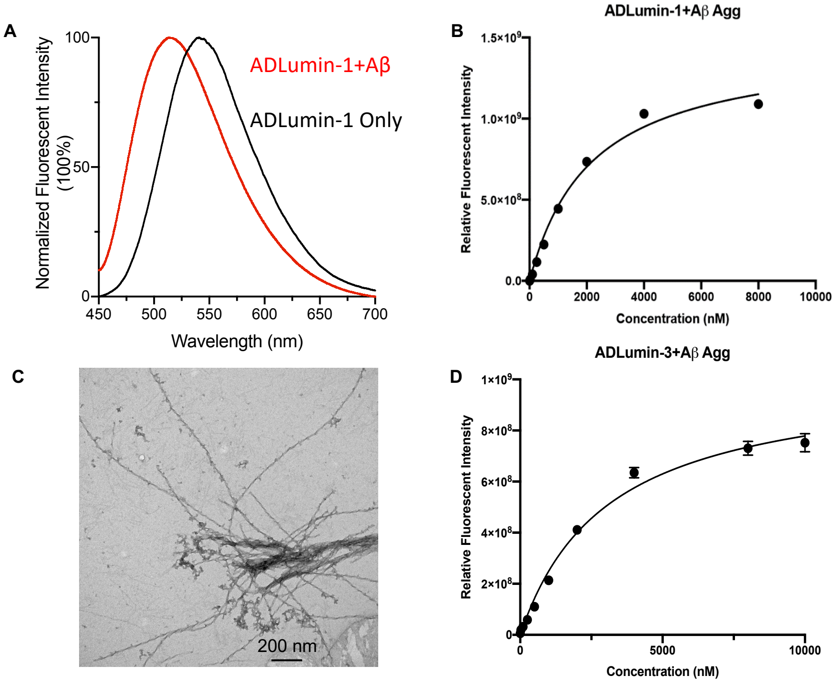

Supplementary Figure 2 Kd measurement of ADLumin-1 and ADLumin-3 in the presence of Aβ aggregates. A) Fluorescence spectra of ADLumin-1 before and after mixing with Aβ40 aggregates; B) Binding affinity assay between Aβ40 aggregates and ADLumin-1. The fluorescence intensities of ADLumin-1 (250 nM) at 515 nm were measured with increasing concentration of Aβ40 aggregates from 0 to 8 μM. Binding constant K_d was derived from the fitted curve. C) TEM image of Aβ40 aggregates. Data of C were repeated three times independently with similar results. D) Binding affinity assay between Aβ40 aggregates and ADLumin-3. Data point with its error bar indicates mean ± s.d. derived from n=3 biologically independent samples.

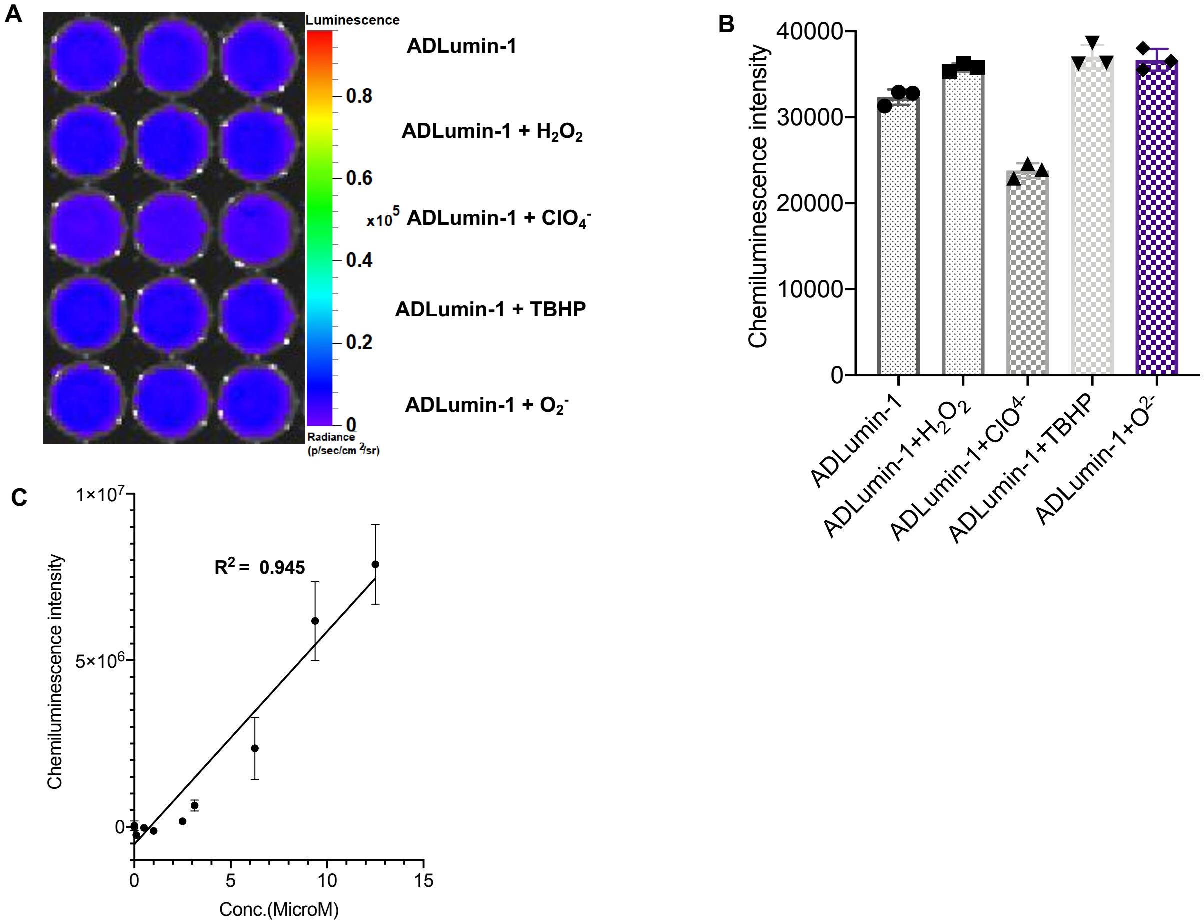

Supplementary Figure 3 Chemiluminescence of ADLumin-1 in the presence of ROS and the linear fitting with Aβs. A) Chemiluminescence of ADLumin-1 after mixing with different ROS species (triplicated); B) Quantitative analysis of the image in (a). There were no significant changes of ADLumin-1 in the presences of the ROS species. C) The linear fitting of Aβ concentrations and the intensity of chemiluminescence in the presence of mouse brain homogenate. Data are represented as mean ± s. d. with n = 3 biologically independent samples.

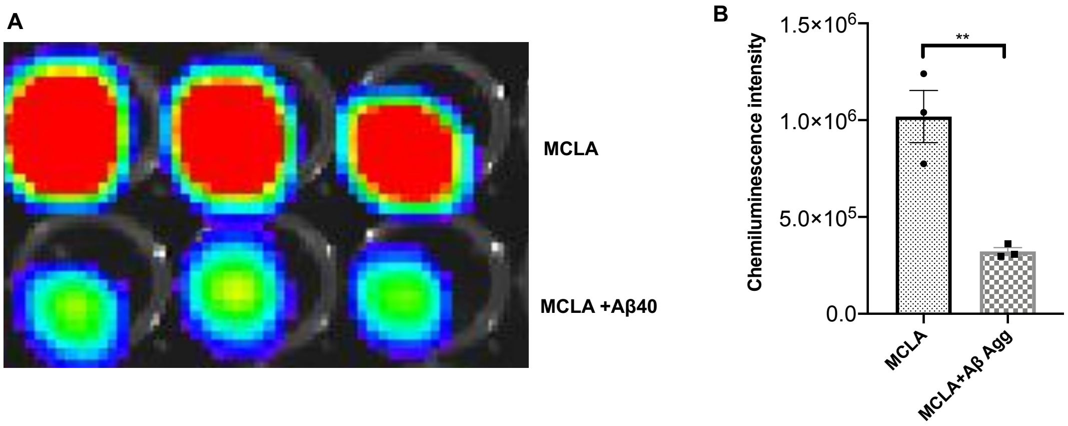

Supplementary Figure 4 Chemiluminescence of MCLA in the presence of Aβs. A) In vitro chemiluminescence images of MCLA (12.5 uM) alone and in the presence of Aβ40 aggregates (12.5 uM) in PBS, pH 7.4; B) Chemiluminescence intensity quantification of image in (A) (mean ± s. d., n=3 biologically independent samples. P = 0.0069. The P-values were generated by Graphpad Prism 8 with two-tailed unpaired *t* test; ** P value < 0.01). Source data underlying B are provided as Source Data file.

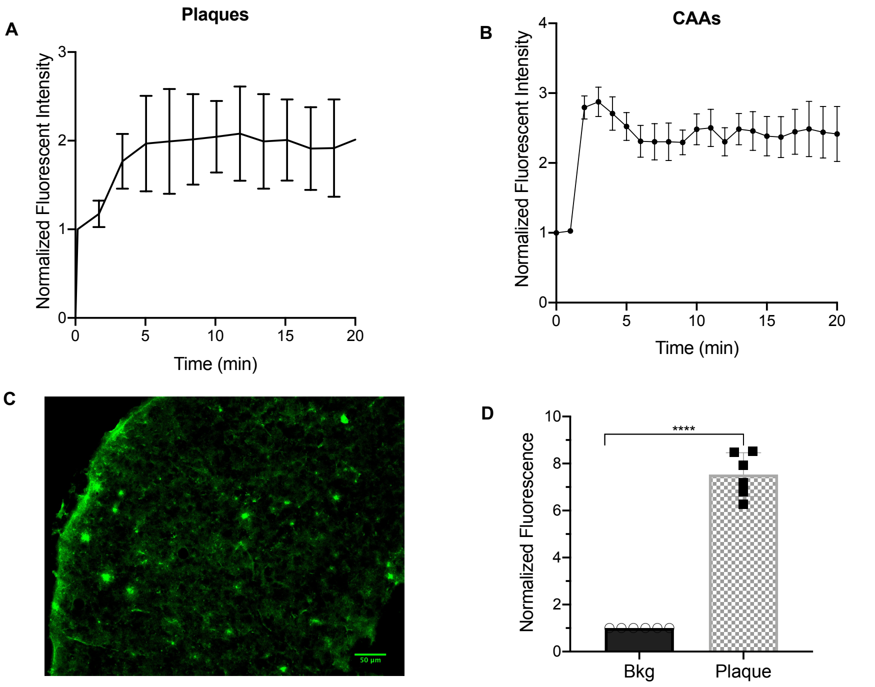

Supplementary Figure 5. In vivo dynamics of ADLumin-1 and in vitro staining with ADLumin-1. A-D) Quantitative analysis of two photon images for plotting the dynamic curves of ADLumin-1 for plaques, and CAAs. The intensity before injection was normalized to 1.0. (mean ± s. d., n=6 biologically independent samples). C) In vitro brain slice imaging with ADLumin-3; D) Quantitative analysis of SNR of plaques in (C). Seven ROIs were averaged (mean ± s. d., n=7 independent meaurements. $P < 0.0001$. The P-values were generated by Graphpad Prism 8 with two-tailed unpaired *t* test; **** P value < 0.0001). Source data underlying D are provided as Source Data file.

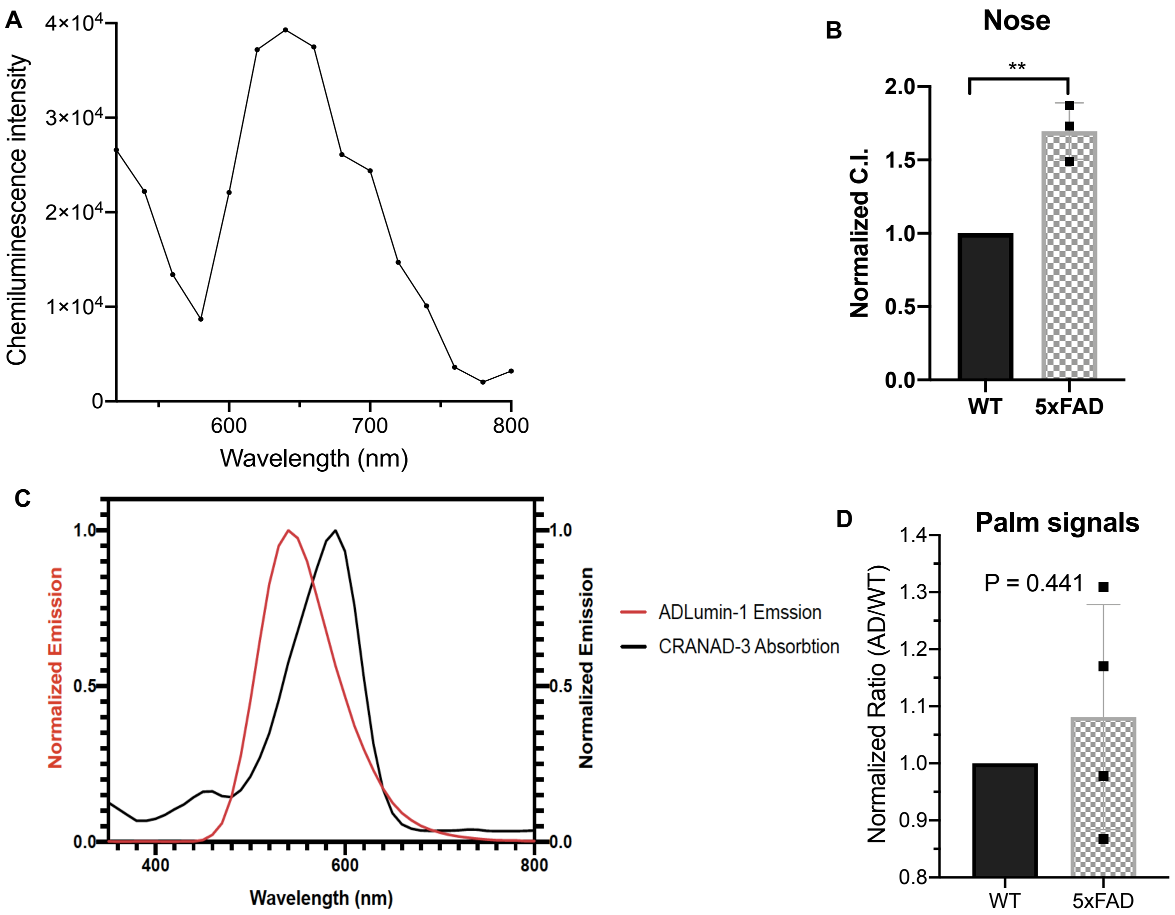

Supplementary Figure 6 Spectra of ADLumin-1 and CRANAD-3 and quantification of in vivo imaging signal of ADLumin-1 from nose and palms. A) In vivo chemiluminescence spectrum of ADLumin-1 from the brain area of a WT mouse. B) Quantitative analysis of nose images from in vivo imaging with ADLumin-1 for 5xFAD and WT; Data points with its error bar indicates mean ± s.d., derived from n = 3 biologically independent animals. P = 0.0033. The P-values were generated by Graphpad Prism 8 with two-tailed unpaired *t* test; ** P value < 0.01. C) Spectral overlap of the ADLumin-1 chemiluminescence emission with the CRANAD-3 absorption. D) Quantitative analysis of palm images from in vivo imaging with ADLumin-1 for 5xFAD and WT. There are no significant differences between the two groups. Data points with its error bar indicates mean ± s.d., derived from n = 4 biologically independent mesurements. The P-values were generated by Graphpad Prism 8 with two-tailed unpaired *t* test. Source data underlying B and D are provided as Source Data file.

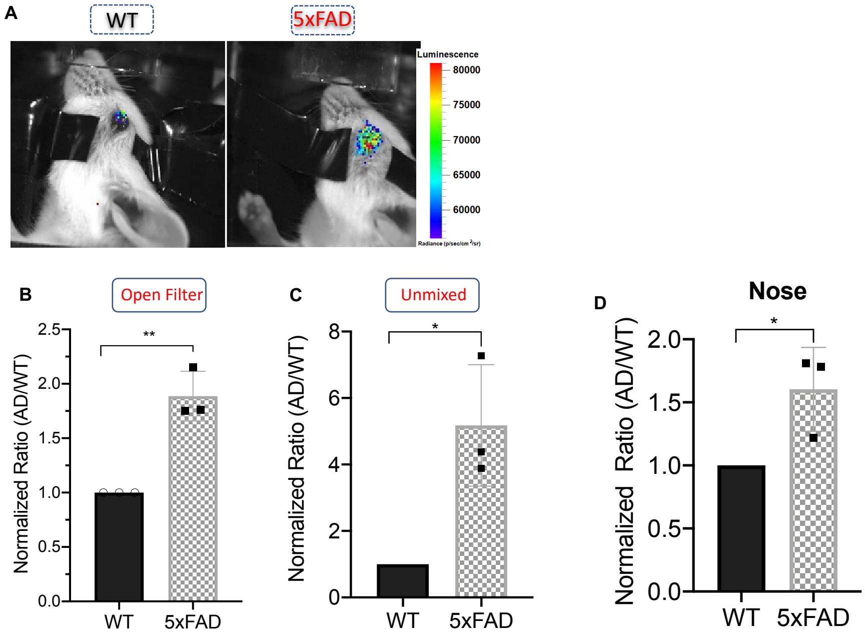

Supplementary Figure 7 In vivo ocular imaging with the CRET pair. A) In vivo chemiluminescence eye imaging of WT and 5XFAD mice with mixture of ADLumin-1 and CRANAD-3; B-C) Quantitative analysis of the images with the setting of open filter and spectral unmixing. D) Quantitative analysis of nose images with the CRET pair. Data are expressed as mean ± s.d. with n=3 biologically independent animals. P = 0.0025 (B); P = 0.0256 (C), and P = 0.0168 (D). The P-values were generated by Graphpad Prism 8 with two-tailed unpaired *t* test; ** P value < 0.01, * P value < 0.05. Source data underlying B, C and D are provided as Source Data file.

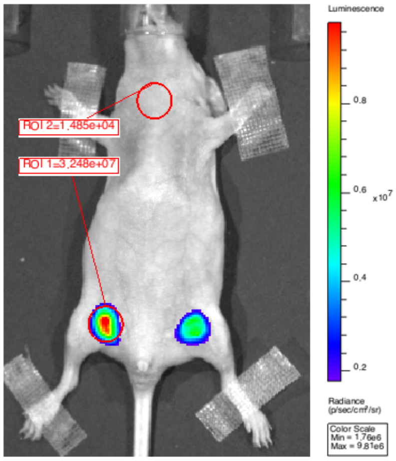

Supplementary Figure 8 ADLumin-1 could prove a very high SNR (>2000). SNR = ROI_1/ROI_2 = 2189.

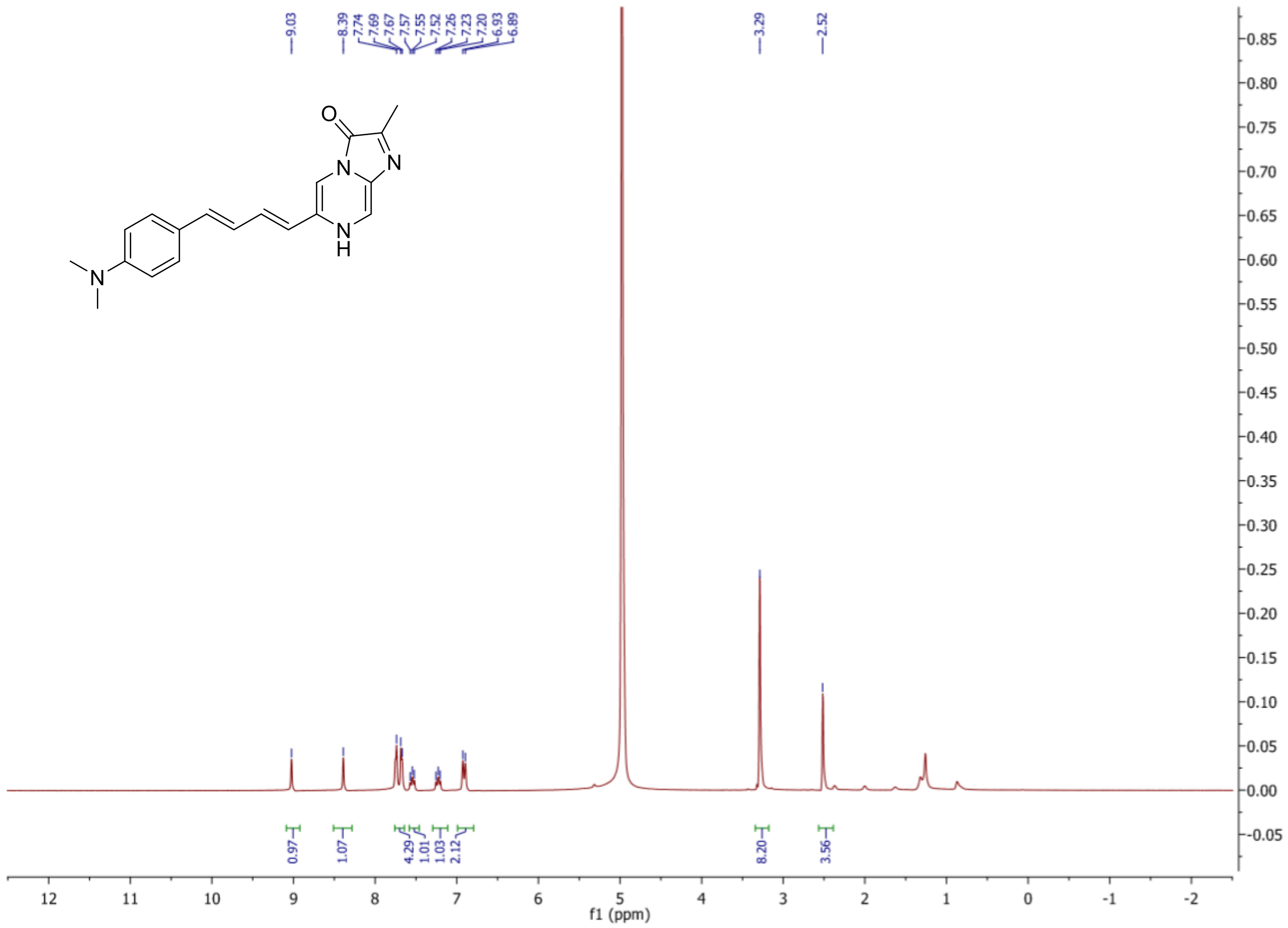

Supplementary Figure 9 ^{1}H-NMR of ADLumin-1

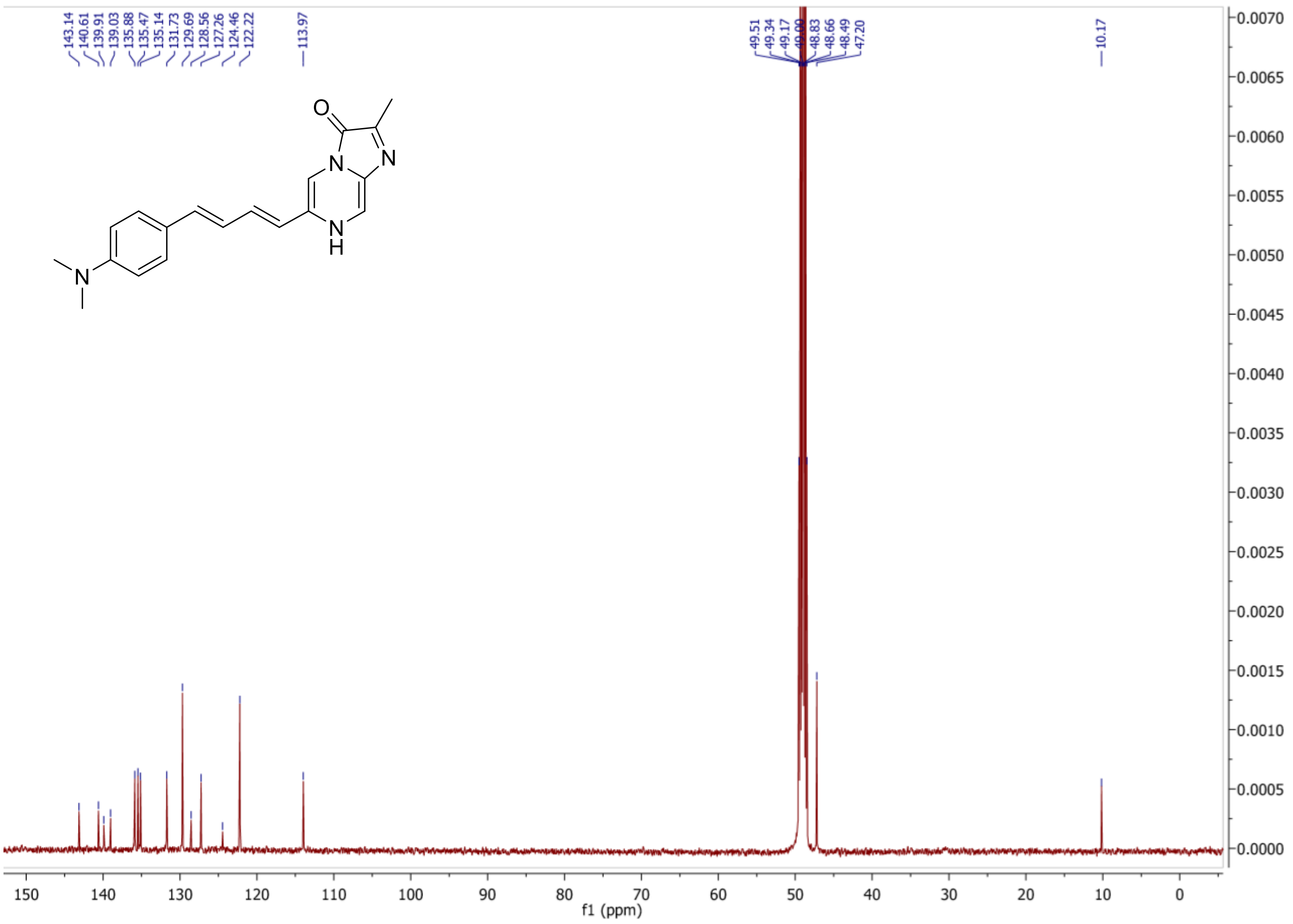

Supplementary Figure 10 ^{13}C-NMR of ADLumin-1

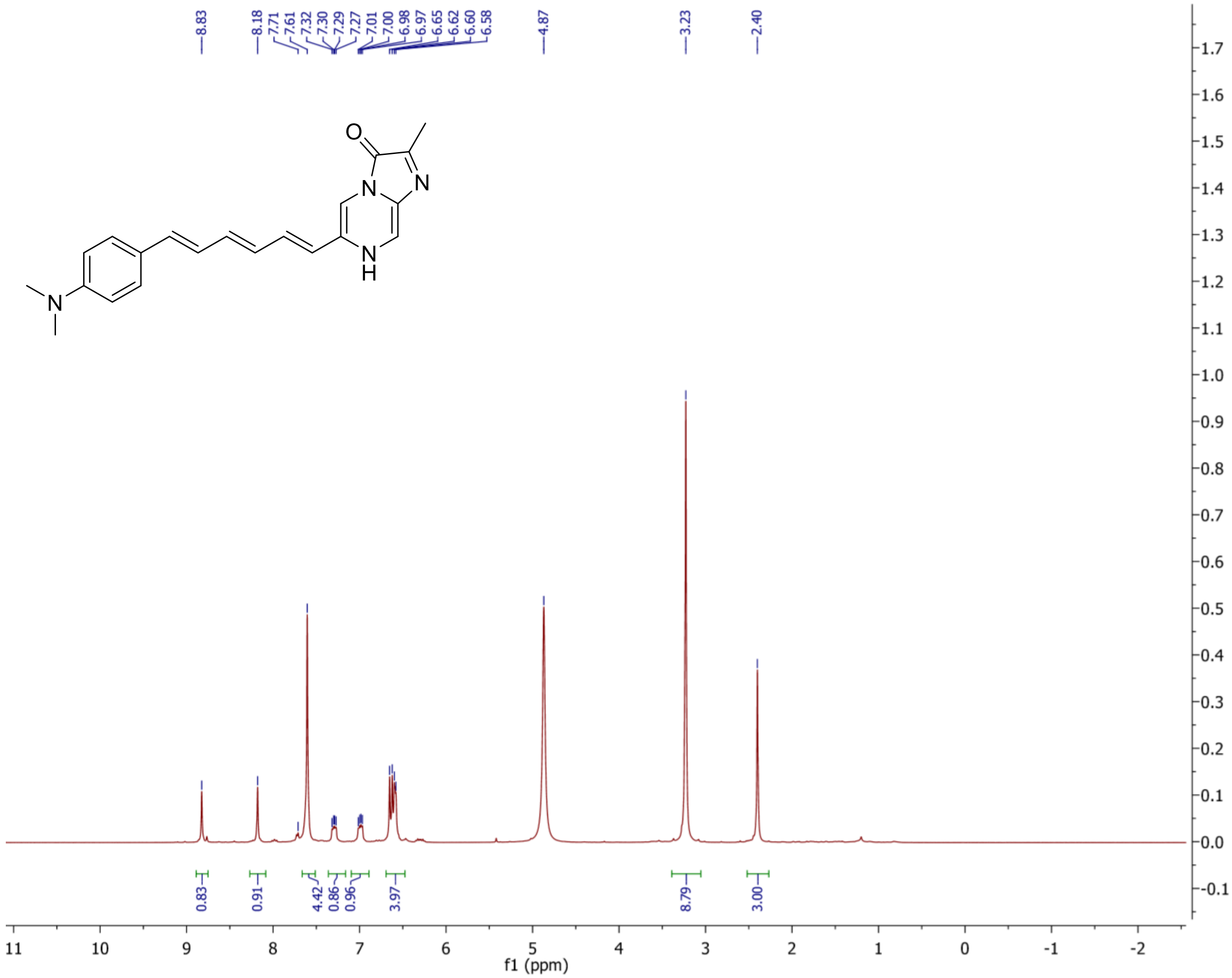

Supplementary Figure 11 ^{1}H-NMR of ADLumin-2

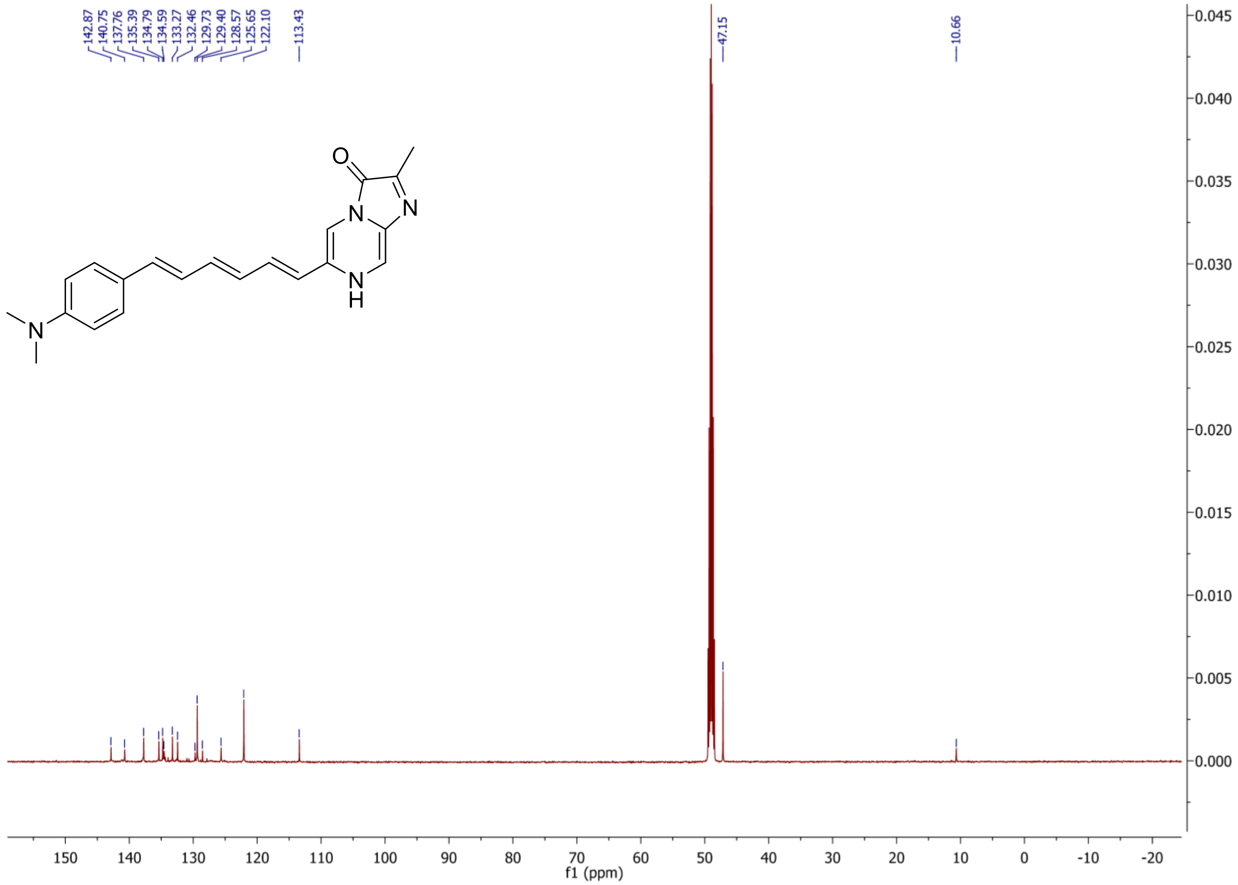

Supplementary Figure 12 ^{13}C-NMR of ADLumin-2

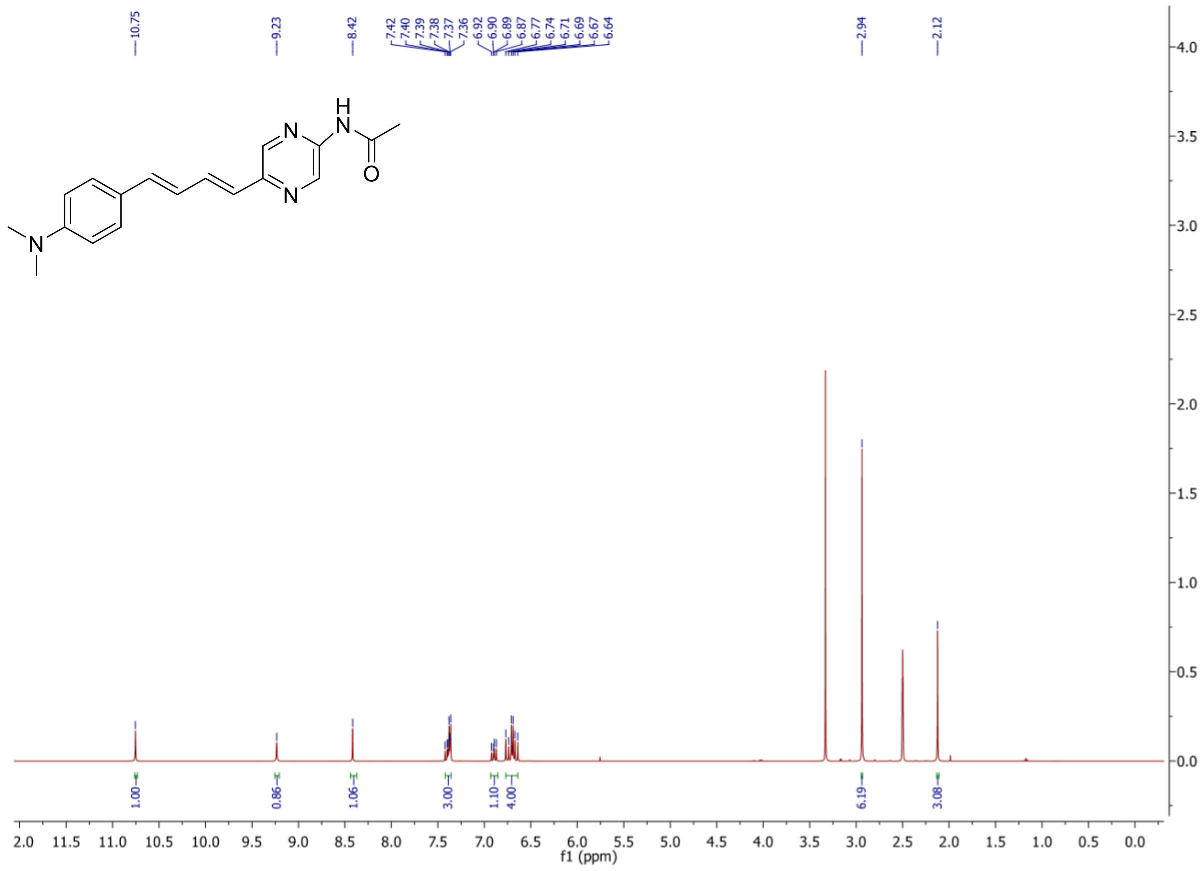

Supplementary Figure 13 ^{1}H-NMR of ADLumin-3

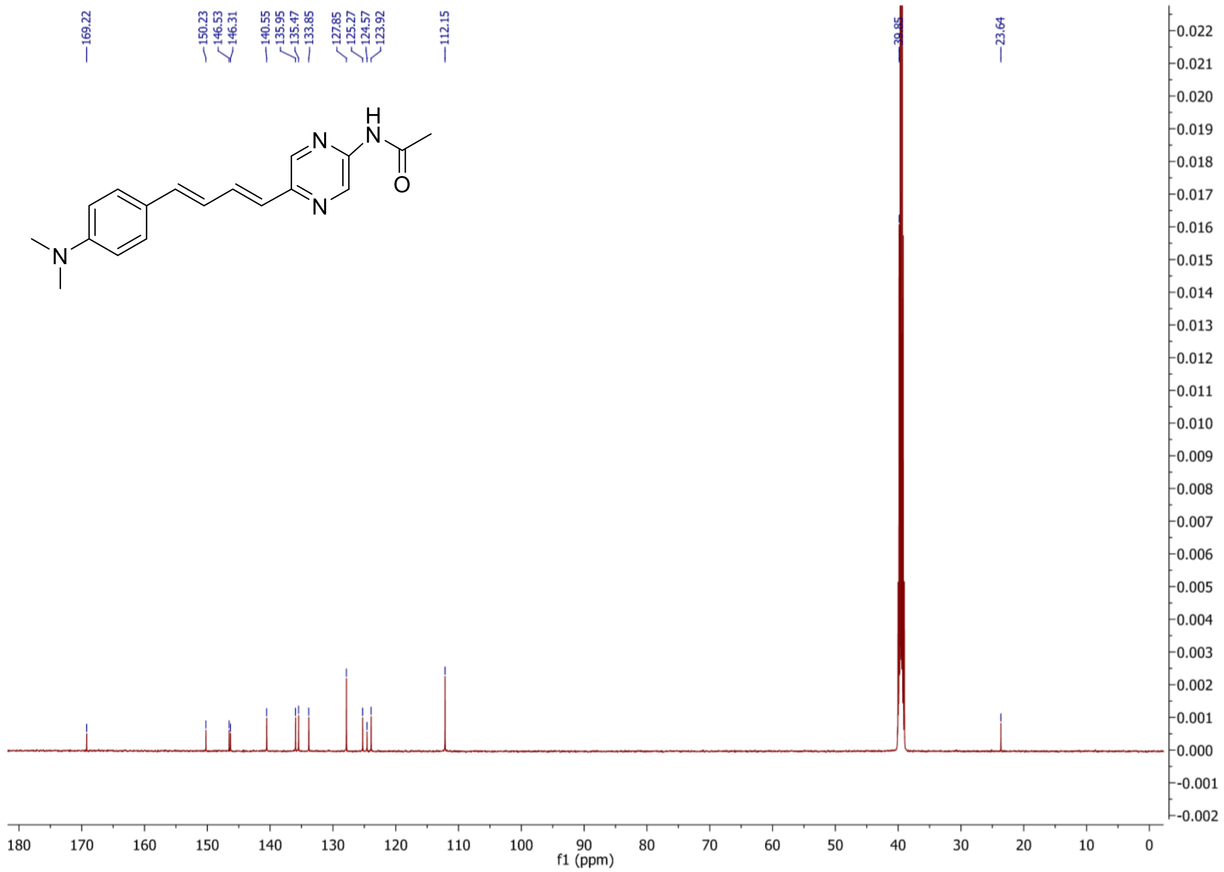

Supplementary Figure 14 ^{13}C-NMR of ADLumin-3
