# Supplementary material for: Turn-on chemiluminescence probes and dual-amplification of signal for detection of amyloid beta species in vivo
Source: Nat Commun. 2020 Aug 13;11:4052. doi: 10.1038/s41467-020-17783-4 (PMC7426431; doi:10.1038/s41467-020-17783-4)
Supplement: Supplementary file 3 — Reporting Summary [file 41467_2020_17783_MOESM3_ESM.pdf]

nature research

Corresponding author(s): Chongzhao Ran

Last updated by author(s): 2020/06/28

Reporting Summary

Nature Research wishes to improve the reproducibility of the work that we publish. This form provides structure for consistency and transparency in reporting. For further information on Nature Research policies, see our Editorial Policies and the Editorial Policy Checklist.

Statistics

For all statistical analyses, confirm that the following items are present in the figure legend, table legend, main text, or Methods section.

n/a	Confirmed	
☐	☒	The exact sample size (*n*) for each experimental group/condition, given as a discrete number and unit of measurement
☐	☒	A statement on whether measurements were taken from distinct samples or whether the same sample was measured repeatedly
☐	☒	The statistical test(s) used AND whether they are one- or two-sided<br>*Only common tests should be described solely by name; describe more complex techniques in the Methods section.*
☒	☐	A description of all covariates tested
☐	☒	A description of any assumptions or corrections, such as tests of normality and adjustment for multiple comparisons
☐	☒	A full description of the statistical parameters including central tendency (e.g. means) or other basic estimates (e.g. regression coefficient) AND variation (e.g. standard deviation) or associated estimates of uncertainty (e.g. confidence intervals)
☐	☒	For null hypothesis testing, the test statistic (e.g. *F*, *t*, *r*) with confidence intervals, effect sizes, degrees of freedom and *P* value noted<br>*Give P values as exact values whenever suitable.*
☒	☐	For Bayesian analysis, information on the choice of priors and Markov chain Monte Carlo settings
☒	☐	For hierarchical and complex designs, identification of the appropriate level for tests and full reporting of outcomes
☒	☐	Estimates of effect sizes (e.g. Cohen's *d*, Pearson's *r*), indicating how they were calculated

Our web collection on statistics for biologists contains articles on many of the points above.

Software and code

Policy information about availability of computer code

Data collection	Imaging data was collected on IVIS Spectrum (Perkin Elmer, MA).
Data analysis	Imaging data was analyzed with IVIS LivingImaging 4.2.1 software that is associated with IVIS Spectrum imaging system, and ImagJ 1.47V.

For manuscripts utilizing custom algorithms or software that are central to the research but not yet described in published literature, software must be made available to editors and reviewers. We strongly encourage code deposition in a community repository (e.g. GitHub). See the Nature Research guidelines for submitting code & software for further information.

Data

Policy information about availability of data

All manuscripts must include a data availability statement. This statement should provide the following information, where applicable:
- Accession codes, unique identifiers, or web links for publicly available datasets
- A list of figures that have associated raw data
- A description of any restrictions on data availability

All data supporting the findings of this study are included in the Article and its Supplementary Information. Source Data for Figs. 2B, C, 3E, G, 4B, 5D, E-F, 6B, D, 9C, D, E, and Supplementary Figs 3B, 4B, 5D, 6B, D, 7B, C, Dare available with this paper.

Field-specific reporting

Life sciences study design

All studies must disclose on these points even when the disclosure is negative.

Sample size	No Sample size calculation was performed. The size number is how many samples used for the experiments. We determined the sizes based on whether minimal n=3 or n=4 could reach p value <0.05.
Data exclusions	No exclusion of data was involved for in vitro experiments. For in vivo mouse studies, data was excluded if the injection was bad.
Replication	All experiments are replicable according to the described procedures in the manuscript. Unless explicitly stated, all data shown were obtained from at least 3 biological independent experiments. For representative images, each experiment was successfully repeated at least three times under similar conditions.
Randomization	No randomization was performed, due to our studies are for imaging method validation.
Blinding	No blinding studies was performed, due to our studies are imaging method validation.

Reporting for specific materials, systems and methods

We require information from authors about some types of materials, experimental systems and methods used in many studies. Here, indicate whether each material, system or method listed is relevant to your study. If you are not sure if a list item applies to your research, read the appropriate section before selecting a response.

Materials & experimental systems

n/a	Involved in the study	
☒	☐	Antibodies
☒	☐	Eukaryotic cell lines
☒	☐	Palaeontology and archaeology
☐	☒	Animals and other organisms
☒	☐	Human research participants
☒	☐	Clinical data
☒	☐	Dual use research of concern

Methods

n/a	Involved in the study	
☒	☐	ChIP-seq
☒	☐	Flow cytometry
☒	☐	MRI-based neuroimaging

Animals and other organisms

Policy information about studies involving animals; ARRIVE guidelines recommended for reporting animal research

Laboratory animals	B6SJL-Tg(APPSwFlLon,PSEN1*M146L*L286V)6799Vas/Mmjax, MMRRC Stock No: 34840-JAX | 5XFAD. 4-month old, Female.
Wild animals	No wild animals were used in this study
Field-collected samples	No field collected samples were used in the study
Ethics oversight	All animal experiments were approved by the Institutional Animal Use and Care Committee at Massachusetts General Hospital.

Note that full information on the approval of the study protocol must also be provided in the manuscript.
